# The use of Goal Attainment Scaling in a community health promotion initiative with seniors

**DOI:** 10.1186/1471-2318-7-16

**Published:** 2007-07-03

**Authors:** Marita Kloseck

**Affiliations:** 1 Faculty of Health Sciences, The University of Western Ontario, Arthur and Sonia Labatt Health Sciences Building, Room 216, London, Ontario, N6A 5B9, Canada

## Abstract

**Background:**

Evaluating collaborative community health promotion initiatives presents unique challenges, including engaging community members and other stakeholders in the evaluation process, and measuring the attainment of goals at the collective community level. Goal Attainment Scaling (GAS) is a versatile, under-utilized evaluation tool adaptable to a wide range of situations. GAS actively involves all partners in the evaluation process and has many benefits when used in community health settings.

**Methods:**

The purpose of this paper is to describe the use of GAS as a potential means of measuring progress and outcomes in community health promotion and community development projects. GAS methodology was used in a local community of seniors (n = 2500; mean age = 76 ± 8.06 SD; 77% female, 23% male) to a) collaboratively set health promotion and community partnership goals and b) objectively measure the degree of achievement, over- or under-achievement of the established health promotion goals. Goal attainment was measured in a variety of areas including operationalizing a health promotion centre in a local mall, developing a sustainable mechanism for recruiting and training volunteers to operate the health promotion centre, and developing and implementing community health education programs. Goal attainment was evaluated at 3 monthly intervals for one year, then re-evaluated again at year 2.

**Results:**

GAS was found to be a feasible and responsive method of measuring community health promotion and community development progress. All project goals were achieved at one year or sooner. The overall GAS score for the total health promotion project increased from 16.02 at baseline (sum of scale scores = -30, average scale score = -2) to 54.53 at one year (sum of scale scores = +4, average scale score = +0.27) showing project goals were achieved above the expected level. With GAS methodology an amalgamated score of 50 represents the achievement of goals at the expected level.

**Conclusion:**

GAS provides a "participatory", flexible evaluation approach that involves community members, research partners and other stakeholders in the evaluation process. GAS was found to be "user-friendly" and readily understandable by seniors and other community partners not familiar with program evaluation.

## Background

Ongoing funding constraints, health system restructuring and the steadily growing impact of chronic diseases have led to major changes in the delivery of health care services. In particular, there has been an increased emphasis on active and healthy aging [1,2], community health services and supports [3], and community collaboration around health issues with a particular emphasis on self-help models of community development [4-7]. Along with these changing trends is the ever growing need to demonstrate value for money. A solid evaluation framework is increasingly being expected by funders who want to see that community project funds are well spent. It is therefore critical to ensure that community health promotion initiatives are conducted with well-planned evaluation strategies able to demonstrate changes and outcomes achieved. With the current focus on evidence-based and collaborative practices much emphasis has been placed on participatory evaluation methods [7-10]. Thus it is surprising that many health-related community development initiatives continue to lack a scientific evaluation framework, continue to rely on anecdotal reports of program activities and successes, fail to use "participatory" evaluation processes, and still do not set clear and measurable goals to evaluate the impact of their activities [7,11,12]. In part, this is a result of the complexity of participatory action research and community health promotion initiatives.

Numerous researchers have outlined process evaluation methodologies suitable for a health promotion context [9,13,14], participatory evaluation approaches [7,10] and health promotion evaluation methods in general [15]. Current commonly used process evaluation methods include surveys and questionnaires for community members and leaders, event logs, activity logs, key informant interviews, focus group methodology, meeting observation and review of existing documents [9]. Chilaka [14] developed a process that uses quantitative computed community participation (Cp) values to measure community engagement. Still others [13] developed scales to measure community leaders' perceptions of collaborative community initiatives, including such things as community participation, community resources, leadership, advocacy, planning, community involvement, communication and creativity.

Thompson [15] provides a comprehensive discussion of program evaluation within a health promotion framework. Specifically, she outlines four major paradigms for evaluation as developed by Smith and Glass [16], these being the randomized controlled trial, the closed system evaluation (e.g., setting project specific goals and measuring their achievement), the professional judgement model (e.g., an accreditation process) and a political model (e.g., stakeholder and funder interests). The first two paradigms are of most concern to us here, although the political needs of the stakeholders can, we believe, be included within the closed system evaluation. One characteristic of community development, whether it is in relation to health promotion or not, is that the outcomes must meet the needs of the specific community. In addition, it is important to measure the success of community development efforts, for example, community capacity building (i.e., enhancing the ability of a community to collectively identify and work toward solutions to it's own problems), as well as the ability of the empowered community to achieve the desired outcomes. One feature of the typical randomized controlled trial is that the outcomes being measured in the intervention and control groups need to be the same, and need to be measured in a standardized, validated way, characteristics which, as pointed out above, may not be compatible with community-identified and community specific processes and outcomes. This makes it difficult to compare processes and outcomes in one community with those in another when the two communities may not be comparable in either assets or objectives. Comparing two approaches to community development would be possible in a randomized trial with communities as the unit of study, and the degree of goal achievement as the outcome. The sample size and the costs, however, would be prohibitive. This is of course also dependent upon the ability to define distinct community development methodologies for comparison.

The closed system evaluation outlined by Thompson [15] appears more feasible and implies the judgement of outcomes against prior established goals which are specific to, and established by, a particular community. The evaluation determines the degree to which the goals are achieved rather than which community development approach was most successful in achieving them. The initiative is judged against the degree of achievement of its own goals. It is this approach to evaluation that we are concerned with here, and the use of one particularly effective evaluation method, Goal Attainment Scaling (GAS), in measuring such achievement. This article will describe the use of GAS [17-21] as a potential means of measuring progress and outcomes in such a multi-faceted and complex situation as community health promotion and community development initiatives.

## Methods

### Community health promotion & community development context

Our health promotion and community development initiative, the *Cherryhill Healthy Aging Program *[5,22] is a participatory action research program designed to foster partnerships among seniors, health professionals and other community members to work collaboratively to develop, implement and evaluate new and innovative strategies to optimize the health, independence and quality of life of older individuals living in the community. The purpose of this paper is not to evaluate the *Cherryhill Healthy Aging Program *as a whole, but rather to use this initiative to illustrate the value of GAS in this type of endeavour.

The Cherryhill community is a compact, high density apartment complex in London, Ontario which consists of 13 private apartment buildings with 2325 units and a total population of 2925 citizens (mean age = 76 years, ± 8.06 years SD). This local seniors' community has a high concentration of older individuals and is an area of high health service utilization. Seventy-seven percent of residents are 65 years of age or older, with the majority elderly women living alone. The community is a stable community with individuals aging in place. Average time lived in this community was 10 years, with the oldest individuals (85+ years) having lived in the community longest (14+years). The *Cherryhill Healthy Aging Program *used a comprehensive and complex evaluation framework that includes a variety of quantitative and qualitative methods, including goal attainment scaling. The *Cherryhill Healthy Aging Program *[5] is an ongoing health promotion initiative in a local community of seniors that is expanding and evolving based on both evidence and community-identified needs. The program was initiated in September 1996 to build capacity for health in a community of seniors. It began as a community-university research partnership. In 2002 the program received annualized funding from the Ontario Ministry of Health and Long-Term Care and numerous community health agency partners, including the Victorian Order of Nurses (VON), Community Care Access Centre for Southwestern Ontario (CCAC), and St. Joseph's Health Care London are now working with community seniors through the Cherryhill Health Promotion and Information Centre located in the Cherryhill Village Mall. While numerous participatory evaluation methods were used to evaluate the *Cherryhill Healthy Aging Program*, GAS methodology proved to be particularly useful.

This paper focuses on the use of GAS to guide and evaluate volunteer recruitment, training and support, and the development of community capacity as the necessary first steps for community-based health promotion initiatives which will be the subject of further reports. The comprehensive evaluation framework of the *Cherryhill Healthy Aging Program *is described in detail elsewhere [5].

### Goal attainment scaling

Goal Attainment Scaling (GAS) [17-21] is a method for setting goals and measuring the degree of goal achievement by creating an individualized 5-point scale (-2, -1, 0, +1, +2) of potential outcomes for each activity undertaken. Each scale is created *de novo *around the expected level of achievement of a particular individual, program or project goal. Above and below this level, indicators of under-achievement and over-achievement (i.e., getting not as far as, or farther than, expected) can be created in order to evaluate the degree of success in achieving the goal. A standardized method of scoring the goal attainment scale, and of amalgamating several goals into one overall score, has been devised and provides a measure of the overall success of the program or project. GAS can be used by professionals and trained members of the general public to measure program or project goals at various levels including process and outcome levels. The advantage of GAS methodology is that it is more a process or method than a tool, and that it is endlessly versatile to meet the needs of each new situation. The disadvantage is that, not being a fixed tool, it lacks the psychometric properties usually expected of assessment and measurement tools. This has been a source of criticism and contention in the past [17,23], but the scale has, nevertheless, acquired acceptance in various clinical fields. In effect, the GAS process allows the creation of a unique tool for each situation, which is the basis of its versatility and its particular value in health promotion and community development. The content of the scale, devised within the health promotion/community development context, is determined by each project and the community members' sense of what within the project is important to them. This can include achievements which would likely be taken into account in a subjective evaluation of the satisfaction, or otherwise, of the activity. GAS can, therefore, also capture the more subjective impressions of success. It was anticipated that GAS would prove useful in evaluating health programs involving the general public in an active role – individuals who are likely to be unfamiliar with scientific methods. It is sufficiently "user-friendly" for older participants of community health promotion programs to use. In addition, it can also accommodate the needs of health promotion staff, community partners, stakeholders, funders and program evaluators, and be used alone or in combination with other quantitative and qualitative methods.

### An overview of the GAS process

The GAS process requires the identification of practical goals expected to be achieved through the project. Once goal areas have been identified for each goal, the required or expected level of achievement of each goal is stated. This is scored as "0" and represents the most probable level of result achieved. An example for one goal is given in Table 1. "Lesser" (score -1 and -2) and "greater" (score +1 and +2) scale levels are then filled in. To allow for a decrease this is typically scored at -1 but as most community development projects are starting de novo the baseline is accordingly scored at -2. With time, the expectation is that the score will move from -2 to 0. Under-achievement of goals may be reflected in a score of -1 whereas goal achievement at the +1 and +2 level will represent greater degrees of success than originally expected. The overall goal will likely be represented by several sub-goals. Table 2 provides an example of the two sub-goals for operationalizing the Health Promotion and Information Centre, of which the example (Table 1) is one. At the end of a community health promotion project scale scores can be summed and the overall GAS score for the project can be calculated using the following formula [17]:

**Table 1:** Example of Goal Attainment Scaling with a single sub-goal of thematic area 1

GOAL ATTAINMENT LEVELS	CHERRYHILL HEALTHY AGING PROGRAM SINGLE GOAL	GAS SCORE
		
	Community Volunteers to Operate the Cherryhill Health Promotion & Information Centre	
Much less than expected -2	*No trained community volunteers (seniors) to operate the Cherryhill Health Promotion & Information Centre. No formal sustainable system for recruiting volunteers. ***a**	30.00
Somewhat less than expected -1	*Some, but not enough, trained community volunteers (seniors) to operate the Cherryhill Health Promotion & Information Centre. No formal sustainable system for recruiting volunteers. Recruitment of volunteers conducted by health professionals. ***b**	40.00
Expected level (Program Goal) 0	*Adequate number of trained community volunteers (seniors) to operate the Cherryhill Health Promotion & Information Centre 6 days per week (Monday to Saturday). Formal sustainable system for ongoing recruitment of volunteers. Recruitment of volunteers conducted by health professionals in collaboration with Cherryhill seniors. ***c, d**	50.00
Somewhat better than expected +1	*Adequate number of trained community volunteers (seniors) to operate the Cherryhill Health Promotion & Information Centre 6 days per week (Monday to Saturday). Formal sustainable system for ongoing recruitment of volunteers. Recruitment of volunteers conducted by Cherryhill seniors in collaboration with health professionals. ***e**	60.00
Much better than expected +2	*Adequate number of trained community volunteers (seniors) to operate the Cherryhill Health Promotion & Information Centre 6 days per week (Monday to Saturday). Formal sustainable system for ongoing recruitment of volunteers. Recruitment of volunteers conducted by Cherryhill seniors. ***f**	70.00

Goal Status Baseline: **a**

3 Months: **b**

6 Months: **c**

9 Months: **d**

12 Months: **e**

24 Months: **f**

**Table 2:** Goal Attainment Scaling with the two sub-goals of thematic area 1

GOAL ATTAINMENT LEVELS	OPERATIONALIZING A HEALTH PROMOTION & INFORMATION CENTRE IN THE CHERRYHILL COMMUNITY MALL	
	
	Community Volunteers to Operate the Cherryhill Health Promotion & Information Centre	Provision of Health Information & Education Displays in the Health Promotion Centre
Much less than expected -2	*No trained community volunteers (seniors) to operate the Cherryhill Health Promotion & Information Centre. No formal sustainable system for recruiting volunteers. ***a**	*No health information or education displays. ***a**
Somewhat less than expected -1	*Some, but not enough, trained community volunteers (seniors) to operate the Cherryhill Health Promotion & Information Centre. No formal sustainable system for recruiting volunteers. Recruitment of volunteers conducted by health professionals. ***b**	*Health information & 1–2 health education displays in the Cherryhill Health Promotion & Information Centre per year in Cherryhill mall; topics identified & organized by health professionals in collaboration with community volunteers. ***b, c, d**
Expected level (Program Goal) 0	*Adequate number of trained community volunteers (seniors) to operate the Cherryhill Health Promotion & Information Centre 6 days per week (Monday to Saturday). Formal sustainable system for ongoing recruitment of volunteers. Recruitment of volunteers conducted by health professionals in collaboration with Cherryhill seniors. ***c, d**	*Health information & 3–4 health education displays in the Cherryhill Health Promotion & Information Centre per year in Cherryhill mall; topics identified & organized by health professionals in collaboration with community volunteers. ***e, f**
Somewhat better than expected +1	*Adequate number of trained community volunteers (seniors) to operate the Cherryhill Health Promotion & Information Centre 6 days per week (Monday to Saturday). Formal sustainable system for ongoing recruitment of volunteers. Recruitment of volunteers conducted by Cherryhill seniors in collaboration with health professionals. ***e**	*Health information &*≥ *5 health education displays in the Cherryhill Health Promotion & Information Centre per year in Cherryhill mall; community-identified topics; displays organized by community volunteers in collaboration with health professionals. *
Much better than expected +2	*Adequate number of trained community volunteers (seniors) to operate the Cherryhill Health Promotion & Information Centre 6 days per week (Monday to Saturday). Volunteers include Cherryhill residents. Formal sustainable system for ongoing recruitment of volunteers. Recruitment of volunteers conducted by Cherryhill seniors. ***f**	*Health information &*≥ *5 health education displays in Cherryhill mall with in-reach into Cherryhill apartment buildings; community-identified topics; displays organized by community volunteers in collaboration with health professionals. *
Comments		
Goal Status	Baseline: **a** 3 Months: **b** 6 Months: **c** 9 Months: **d** 12 Months: **e** 24 Months: **f**	Baseline: **a** 3 Months: **b** 6 Months: **c** 9 Months: **d** 12 Months **e** 24 Months: **f**

GAS score=50+10Σ(wixi)√(.7Σwi 2)+.3(Σwi)2
 MathType@MTEF@5@5@+=feaafiart1ev1aaatCvAUfKttLearuWrP9MDH5MBPbIqV92AaeXatLxBI9gBamXvP5wqSXMqHnxAJn0BKvguHDwzZbqegyvzYrwyUfgarqqtubsr4rNCHbGeaGqiA8vkIkVAFgIELiFeLkFeLk=iY=Hhbbf9v8qqaqFr0xc9pk0xbba9q8WqFfeaY=biLkVcLq=JHqVepeea0=as0db9vqpepesP0xe9Fve9Fve9GapdbaqaaeGacaGaaiaabeqaamqadiabaaGcbaGaee4raCKaeeyqaeKaee4uamLaeeiiaaIaee4CamNaee4yamMaee4Ba8MaeeOCaiNaeeyzauMaeyypa0JaeGynauJaeGimaaJaey4kaSYaaSaaaeaacqaIXaqmcqaIWaamcqqHJoWucqGGOaakcqqG3bWDdaWgaaWcbaGaeeyAaKgabeaakiabbIha4naaBaaaleaacqqGPbqAaeqaaOGaeiykaKcabaaccaGae8NgIyTae8hkaGIae8Nla4Iae83naCJaeu4OdmLaee4DaC3aa0baaSqaaiabbMgaPbqaaiabbccaGiabbkdaYaaakiabcMcaPiabgUcaRiabc6caUiabiodaZiabcIcaOiabfo6atjabbEha3naaBaaaleaacqqGPbqAaeqaaOGaeiykaKYaaWbaaSqabeaacqaIYaGmaaaaaaaa@6C46@

In this formula, if all goals are achieved (i.e., final score of "0" for each), the final summated score derived from this formula will be 50. If, on average, the sub-goals are overachieved (i.e., some at the +1 or +2 level), the final summated score will be greater than 50. Conversely, a final score of less than 50 will represent a shortfall in the project's achievements. W_i _is the weighting given to the ith goal and x_i _is the level or numerical score (-2, -1, 0, +1, +2) of the ith goal. Goal achievement should be routinely evaluated and recorded at the predetermined time intervals. Goals may be differentially weighted according to their perceived importance or left unweighted, and the goals of different sub-projects may be summed to create one overall GAS score [20]. However, for simplicity sake, Kiresuk and colleagues [17] provide tables from which the GAS score (referred to as the T-Score) can easily be derived.

### Use of GAS in the Cherryhill Healthy Aging Program

The overall goal of creating the *Cherryhill Healthy Aging Program *and building capacity for health in a local seniors' community was identified by the research team. Sub-goals which provide the necessary building blocks for the overall goal were collaboratively defined by the research team and a committee of 10 community members (seniors) in the Cherryhill community in London, Ontario who agreed to participate. Four key thematic areas were identified i) operationalizing a Health Promotion and Information Centre, ii) developing a sustainable community volunteer program, iii) building individual and community capacity to identify and resolve community issues, and iv) developing community prevention and health promotion program. Sub-goals were created for each key thematic area as follows:

▪ ***Thematic Area 1*: ***Creating a Health Promotion and Information Centre in the Cherryhill Community *included operationalizing the centre with trained community volunteers and providing health information and education displays in the Health Promotion Centre.

▪ ***Thematic Area 2*: ***Developing a Sustainable Community Volunteer Program *included volunteer recruitment, volunteer training, volunteer support/recognition and volunteer program evaluation.

▪ ***Thematic Area 3*: ***Building Individual and Community Capacity *included creating a formal residents' association, training community volunteers to work effectively as a group, building capacity to identify and resolve community issues and address the community-identified issues of speeding traffic and poor mailbox access as demonstration of community capacity demonstration.

▪ ***Thematic Area 4*: ***Developing a Community Prevention and Health Promotion Program *included developing and implementing a community resident safety check program, developing and implementing a community response team, training seniors to be advocates/risk assessors and developing a system of informal community support ("seniors on call") [Table 3].

**Table 3:** Thematic area and overall goal achievement for the Cherryhill Healthy Aging Program (total no. of sub-goals = 15)

	**Thematic Area 1**		
	*Operationalizing a Health Promotion&Information Centre (2 summed sub-goals)*		
**Time Interval**	**Sum of Scale Scores**	**Average Scale Score**	**GAS Score**

GAS Score-Baseline	- 4	-2.00	25.19
GAS Score-3 Months	- 2	-1.00	37.59
GAS Score-6 Months	-1	-0.50	43.79
GAS Score-9 Months	-1	-0.50	43.79
GAS Score-12 Months	+1	+0.50	**56.21**^b^

	**Thematic Area 2:**		
	*Sustainable Community Volunteer Program (4 summed sub-goals)*		

**Time Interval**	**Sum of Scale Scores**	**Average Scale Score**	**GAS Score**

GAS Score-Baseline	-8	-2.00	20.98
GAS Score-3 Months	-3	-0.75	39.12
GAS Score-6 Months	-2	-0.50	42.75
GAS Score-9 Months	0	0	**50.00**^a^
GAS Score-12 Months	0	0	50.00

	**Thematic Area 3**		
	*Individual & Community Capacity Building (5 summed sub-goals)*		

**Time Interval**	**Sum of Scale Scores**	**Average Scale Score**	**GAS Score**

GAS Score-Baseline	-10	-2.00	19.85
GAS Score-3 Months	-4	-0.80	37.94
GAS Score-6 Months	-2	-0.40	43.97
GAS Score-9 Months	-1	-0.20	46.98
GAS Score-12 Months	+3	+0.60	**59.05**^b^

	**Thematic Area 4**		
	*Community Prevention & Health Promotion Programs (4 summed sub-goals)*		

**Time Interval**	**Sum of Scale Scores**	**Average Scale Score**	**GAS Score**

GAS Score-Baseline	-8	-2.00	20.98
GAS Score-3 Months	-5	-1.25	31.86
GAS Score-6 Months	-2	-0.50	42.75
GAS Score-9 Months	0	0	**50.00**^a^
GAS Score-12 Months	0	0	50.00

Note: ^a ^goals achieved

^b ^goals over-achieved

All goals were unweighted

Under each of the thematic areas, for each sub-goal, a statement which describes each goal's expected achievement (score = 0) and a statement which describes the current status in this goal (score = -2) were agreed upon and the remaining levels of goal achievement representing under-achievement (-1) or over achievement (+1, +2) were defined and recorded on the GAS form. This was done collaboratively by the research team with community members for each sub-goal. Timelines for evaluation were then determined. Three monthly evaluation points were agreed upon. Letters were selected to record baseline (a) and final (f) goal status, and interim progress toward goals (b,c, d, e). These markers indicate the sequence of goal assessment and scoring. At pre-determined time intervals progress was reviewed, the current level of each goal agreed upon, and the appropriate symbol recorded. At each evaluation point the researcher-community committee once again decided whether or not sufficient progress had been made for each goal that the next level of goal achievement could be accepted. A "comments" section allows documentation of circumstances influencing goal progression. At the one year evaluation point goal scores were summed and an overall GAS score was created. At the simplest level the GAS goal forms visually highlight rate of progress. "Red flag" areas that require attention are immediately evident at a glance being shown as clustering of letters or symbols in one box, indicating a stalling of that goal (Table 2). The GAS formula can be used to amalgamate several goals into one overall score to provide a measure of the overall success of the community health promotion initiative.

GAS methodology was used to set goals and evaluate progress toward goals in each of the key thematic areas outlined above. The purpose of this paper is to show how GAS can be used to track the achievement of one goal, show progress within a thematic area containing two goals, and show progress within a program containing four thematic areas. A detailed description of the entire *Cherryhill Healthy Aging Program *is outlined elsewhere [5].

## Results

### Feasibility

GAS was found to be "user-friendly" and easily understood by seniors and other community partners not familiar with program evaluation. At the beginning of the project the process of goal setting and development of GAS forms required approximately 40 minutes per sub-goal and, with experience, time to set goals and complete GAS forms was reduced.

### GAS – achievement, over- and/or under-achievement of goals

Table 1 demonstrates goal progress for one sub-goal of Thematic Area 1. The "a" indicates the starting point at -2 (GAS score 30.00) and it can be seen that the expected level "0" (GAS score 50.00) representing goal achievement was reached by 6 months. This was sooner than expected and further progress was made to +1 (GAS score 60.00) at 1 year and to +2 (GAS score 70.00) by 2 years, representing a level of achievement that was "much better than expected". The overall GAS score for Thematic Area 1, which measured progress related to operationalizing a Health Promotion and Information Centre, is shown in Table 2. The sum of GAS scale scores increased from 25.19 at baseline to 56.21 (over achieved) at 12 months and showed further improvement (GAS score 62.41) at 24 months. Table 3 summarizes the results for the program's four key thematic areas containing a total of 15 sub-goals. The table shows the change in GAS scores across time over a period of 12 months and shows how the rate of goal achievement can vary in different thematic areas. The progress of the *Cherryhill Healthy Aging Program *as a whole can be demonstrated by summing all 15 sub-goals at baseline and at 12 months according to the GAS formula [17] outlined above. The computed baseline GAS score for the total program was 16.02 (sum of scale scores = -30, average scale score = -2.00) and improved to 54.53 at 12 months (sum of scale scores = +4, average scale score = +0.27, median = 0; mode = 0) showing overall goal achievement above the expected level.

## Discussion

Health promotion and health-related community development programs represent a level of complexity that provides a challenge to anyone interested in outcome measurement. By their nature they have a purpose which can be very "personal", reflecting the specific concerns of the community in question, and different from those of other communities. Health promotion initiatives lend themselves best to an internal evaluation, the closed system evaluation as outlined by Thompson [15]. Because of this, GAS methodology, which is particularly well suited to evaluating outcomes which are very individualized, has many benefits in evaluating community projects. This paper attempts to demonstrate the versatility of GAS in defining and tracking goals in evaluating both the process and the outcomes in a community development project. Furthermore, the methodology, by its use of a dimensionless scale, allows the amalgamation of scores across disparate activities, provided there is a logical connection between the activities and they can be seen as reflecting aspects of a common construct.

GAS has been used in different settings to evaluate clinical health program outcomes [18,19], being employed to assess the level of improvement shown by individual clients of a particular program, with the amalgamation of individual client changes being used to evaluate the overall program. The present use of GAS to evaluate the degree of change shown by a *community program as a whole*, as a way of evaluating a community health promotion initiative, is a novel use of this methodology. It is an approach that is flexible, highly versatile, and well suited for health program evaluation with elderly individuals in community development settings. There is, however, little documentation of GAS procedures being used in health promotion or health-related community development settings even though this approach is consistent with, and enhances current empowerment evaluation strategies [8] outlined in evaluation and community development literature.

One challenge with GAS is skilful setting of goals so that goals are neither too easily attained, thereby inviting over-achievement, or alternatively, set so high that goals cannot be achieved. In contrast to the clinical situation, goal achievement in the community may not be a one-time effort as unlike patients in a clinical setting the community doesn't get "discharged" at the end of their length of stay. If goals are easily achieved or over-achieved, new and more ambitious goals can be set and strived for. We don't see this as a short-coming of GAS in the community, and in fact this can be seen as a strength as it allows the community to achieve a sense of success and confidence which can take them forward to the next and more ambitious goals.

There is an ever increasing emphasis on demonstrating that investments produce value for money, particularly in the area of health care where external funding is required. Funders increasingly need to be shown that what they are funding works. No evaluation method will provide validation for a poorly conceived project. One advantage of goal setting is that it has to be done at the outset and can receive the support of all community partners and stakeholders. Agreement on the outcomes of value to the community, the staff and the funders, and the incorporation of these outcomes within the evaluation framework can begin to meet the needs of all involved. The versatility of GAS is such that it can incorporate goals of all types. The emphasis has to be on the logic of the structure designed. For example, goals can be hierarchical such that one may need to be achieved before another is embarked upon. Setting goals in such a manner has, we have found, helped in both the design and implementation of the project, as it can serve as a check on the logic of the approach. A further benefit of GAS is that it is intuitively not difficult and can be readily understood by those with little or no training or experience in evaluation methodology. We have found the lay community members able to understand the concept and process and able to contribute to it. This ensures the ongoing involvement of the community members in both the establishment and scoring of the goals, elucidates the logical structure of both the evaluation and the project itself, and helps obtain support for the whole process.

One of the strengths of GAS is that funders can be involved in the goal setting and goal judging process. Usually in GAS the goal setters are in fact the judgers of goal achievement. It would be possible however for a "blinded" third party to do the goal attainment scoring, thereby reducing any potential bias. The use of GAS does not preclude the use of other more conventional tools, processes and analyses. Indeed, the outcome of these can be incorporated into the GAS system. Many projects contain sub-projects which are more suited to standard methodology, usually of the quantitative type. Within the *Cherryhill Healthy Aging Program*, for example, a study of factors important in falls in this community is one of many sub-projects and research projects that are underway. This requires the use of formal research methodology, sample size estimates and multi-variate analysis. Survey methodology is commonly used in community work and again requires appropriate expertise in survey design and sampling. The process of more formal research endeavours within the community can be embraced within the GAS format. This helps keep community members involved in the project. The process of reviewing progress at timed intervals allows for the identification of goal areas which are stalling and to proactively identify obstacles that require resolution. The advantage of GAS is, however, that it allows the whole project to be covered, including the development of community capacity, the process of implementation and outcome measurement, all of which can be addressed within a common and intuitively understandable framework.

## Conclusion

Many benefits of using GAS have been demonstrated through the *Cherryhill Healthy Aging Program*, a health promotion initiative in a local seniors' community. The greatest benefits of GAS are that it is easy to use by general community members in collaboration with professionals; that it provides a clear measure of goals achieved, and the over- and under-achievement of project goals; that goal achievement is quantifiable and the scores for multiple goals can be amalgamated into one overall, summary score that measures the degree to which a particular project is achieving what it set out to do.

## Competing interests

The author(s) declare that they have no competing interests.

## Pre-publication history

The pre-publication history for this paper can be accessed here:


